# General Practitioners’ Decision Making about Primary Prevention of Cardiovascular Disease in Older Adults: A Qualitative Study

**DOI:** 10.1371/journal.pone.0170228

**Published:** 2017-01-13

**Authors:** Jesse Jansen, Shannon McKinn, Carissa Bonner, Les Irwig, Jenny Doust, Paul Glasziou, Katy Bell, Vasi Naganathan, Kirsten McCaffery

**Affiliations:** 1 Screening and Test Evaluation Program (STEP), Sydney School of Public Health, The University of Sydney, Sydney, New South Wales, Australia; 2 Centre for Medical Psychology and Evidence-based Decision-making (CeMPED), The University of Sydney, Sydney, New South Wales, Australia; 3 Faculty of Health Sciences and Medicine, Bond University, Robina, Queensland, Australia; 4 Centre for Education and Research on Ageing (CERA), Ageing and Alzheimer’s Institute, Concord Hospital, The University of Sydney, Sydney, New South Wales, Australia; Vanderbilt University, UNITED STATES

## Abstract

**Background:**

Primary cardiovascular disease (CVD) prevention in older people is challenging as they are a diverse group with varying needs, frequent presence of comorbidities, and are more susceptible to treatment harms. Moreover the potential benefits and harms of preventive medication for older people are uncertain. We explored GPs’ decision making about primary CVD prevention in patients aged 75 years and older.

**Method:**

25 GPs participated in semi-structured interviews in New South Wales, Australia. Transcribed audio-recordings were thematically coded and Framework Analysis was used.

**Results:**

Analysis identified factors that are likely to contribute to variation in the management of CVD risk in older people. Some GPs based CVD prevention on guidelines regardless of patient age. Others tailored management based on factors such as perceptions of prevention in older age, knowledge of limited evidence, comorbidities, polypharmacy, frailty, and life expectancy. GPs were more confident about: 1) medication and lifestyle change for fit/healthy older patients, and 2) stopping or avoiding medication for frail/nursing home patients. Decision making for older patients outside of these categories was less clear.

**Conclusion:**

Older patients receive different care depending on their GP’s perceptions of ageing and CVD prevention, and their knowledge of available evidence. GPs consider CVD prevention for older patients challenging and would welcome more guidance in this area.

## Introduction

Decision making for older adults is complex, especially for those who have multimorbidity. Scientific evidence for treatments and tests in the older population is often limited as this group has traditionally been excluded from clinical trials, and when included they are generally more fit and healthy than older people in the community [1]. Multimorbidity often leads to polypharmacy, which increases the risk of drug-related problems (e.g. adverse drug reactions) [2]. As a result, it is difficult to predict the effect of each individual drug and to compare the overall benefits and harms [3, 4]. In addition, the older population is heterogeneous. Older people vary widely in their health and function, and in their treatment and health outcome preferences [5–7]. General practitioners (GPs) are at the frontline of providing care for older people, and not surprisingly studies have identified numerous challenges [8, 9].

One issue that stands out as particularly complex is decision making about preventive medications in older people [8–10]. This requires weighing up the potential of future benefits, which is difficult to quantify for an individual, against short term risk of harm due to adverse effects of medications in the context of decreasing life expectancy and overall health. In a qualitative study, GPs expressed feeling “under pressure” from clinical guidelines to prescribe preventive medicines despite acknowledging that potential harms of side effects of preventive medication and polypharmacy may outweigh future risk reduction [11].

Medicines for primary CVD prevention are commonly used in older people [12–14] and these medicines are frequently continued until the end of life when they may not be necessary [14, 15]. Most international guidelines for primary CVD prevention encourage the use of 5- or 10 year absolute (or overall, global or combined) CVD risk scores (AR) to target preventive treatment in asymptomatic patients who are at high risk [16]. However, CVD risk prediction models are not well validated in older people [17]. Since AR increases with age, and people at higher risk benefit more in terms of risk reduction, it is reasonable to argue that older people (and especially otherwise healthy older people) have the potential to benefit at least as much from primary CVD prevention as younger people [18, 19], and otherwise healthy older people should not be denied potentially effective preventive medication based on their age alone. However, many commonly used cardiovascular risk prediction models do not allow for competing risks from non-cardiovascular deaths. This may lead to over-estimation of short term cardiovascular risk and the potential benefit from preventive treatment in older people, especially those with a very limited life expectancy. The benefits and harms of cardiovascular disease preventive medication for older people are therefore uncertain [20, 21].

Several principles have been proposed that are potentially helpful in supporting clinician decision making in this arena [22–24]. Key in all of these approaches is optimising care by carefully tailoring treatment to the context and preferences of the individual (older) patient with multimorbidity. However, systematic reviews suggest that these principles are generally poorly applied in guidelines for this group [25–28]. One review looked at primary CVD prevention in older people specifically, and showed that only a handful of clinical practice guidelines adequately addressed evidence about potential benefits and harms of CVD prevention in older people or how to tailor CVD management to multimorbidity and limited life expectancy [25]. If guidance on primary CVD prevention for older people is so limited, how do GPs navigate this complex topic?

This study investigated GPs’ experiences of primary CVD prevention amongst older patients (aged 75+) to identify how they make decisions about the management of CVD risk in this population.

## Methods

### Participants

Twenty-five GPs participated in New South Wales, Australia. Participants were purposively selected to cover a range of characteristics that are known to influence CVD risk management and/or may influence management of older people [29–32]. This included gender, age, years of GP practice, number of patients in practice aged 75 years and older, and use of absolute CVD risk AR assessment. See Table 1.

**Table 1:** GP characteristics.

Characteristic	Category	n
**Sex**	Female	13
	Male	12
**Age (years)**	<40	5
	40–49	1
	50–59	10
	60+	9
**Years of practice**	<10	2
	10–19	4
	20–29	7
	30+	12
**GP role in practice**	Contractor/sessional/retainer/salaried	12
	Partner/principal	13
**Number of GPs in practice**	1–5	15
	6–10	6
	11+	4

### Recruitment

GPs were sent invitation letters through eight Divisions of General Practice. Fifty-seven GPs returned expression of interest forms, of which 25 GPs were enrolled into this study, the remaining GPs were interviewed as part of a study on CVD risk management in the general population (<75 years of age) [33, 34]. Analyses suggested saturation of key themes, so no further recruitment was conducted [35]. Ethics approval for the study was obtained through the Human Research Ethics Committee of the Sydney Local Health District (Protocol No. X11-0200).

### Data collection

Semi-structured interview schedules covering CVD risk assessment and management were developed by the research team, piloted with a convenience sample of GPs, and revised to improve question clarity (see S1 Text). Participants signed a consent form before being interviewed in person or by telephone. Audio-recordings were transcribed verbatim. Two authors trained in public health qualitative methods (JJ, SM) conducted interviews between November 2011 and September 2012.

### Analysis

A Framework Analysis method was used. This is a matrix-based method of thematic analysis involving five steps: 1) familiarisation with the data through reading/coding/discussing initial transcripts (JJ, SM, CB); 2) creating a thematic framework (JJ, SM, CB); 3) indexing—coding remaining transcripts according to the framework, with iterative revision of framework (JJ, SM); 4) charting—themes/quotes summarised in the framework (JJ, SM); 5) mapping and interpretation—framework data were examined within and across themes and participants, summarised (SM), and discussed with all authors [36]. Rigour was addressed by: repeated coding of transcripts by different team members; constant comparison between the existing framework and new data; detailed documentation of the analysis process; and discussion of themes with all authors, including an experienced qualitative researcher (KM), two academic GPs (JD, PG), and a geriatrician (VN) [37].

## Results

Most GPs felt uncertain about CVD risk management in older people, however they varied in: 1) their awareness of (the lack of) evidence for primary CVD prevention in older people, 2) their perceptions of treatment complexity and feasibility in the older patient context, and 3) their ability and confidence to adapt management accordingly. In the following section, we will explain overall patterns relating to GPs decision making about primary prevention of cardiovascular disease in older adults. Table 2 provides a summary of these themes, with illustrative quotes from the data. For evidence relating to GPs’ decision making approaches, please see S1 Table.

**Table 2:** Summary of themes relating to GPs’ decision making about primary prevention of cardiovascular disease in older adults*.

Theme	Illustrative quote
**Applicability of primary CVD prevention guidelines for older people**	
Same guidelines for all ages	"The guidelines for managing cardiovascular risk are all the same regardless of age" (GP47). “If it’s [the AR calculator] actually validated and is actually in here for most of us to use, it actually should be appropriate to use for anyone.” (GP30)
No valid guidelines for older adults	"Well the [CVD risk] calculators don't tend to go over, much over 75, so they don't help very much" (GP51) “I’d follow the guidelines up until the age of 70, that sounds appropriate but I am less confident if they are age 75” (GP20).
Different guidelines for older adults	“Recently there's a suggestion we can accept higher blood pressure, round about 150, in the elderly and perhaps higher cholesterol" (GP27)
**The older patient context**	
Fit versus frail older adults	"Even if they're not very elderly but very frail or have malignant illnesses I would be much less aggressive in the treatment of cardiovascular risk…otherwise I would treat it aggressively, the same if they were 65 or even 55." (GP43) “They got classified as 'old' and you'd have to say well this lady who is acutely ill now actually lives at home and looks after herself… and leads an active life.." (GP23)
Quality of life as treatment goal	"We just try to put more emphasis on how they're feeling and how they function, if they can walk, if they don't feel dizzy, it's the whole thing." (GP30) “If they do get their weight down, if they do start to exercise a bit more than they were or walk or whatever, they are going to be improving their longevity, their fitness to be older; they’re going to have a better quality of life. We may not make their life longer but we might make it have more quality.” (GP12)
Multimorbidity	"So I approach differently people over 75 who have significant co-morbidities. I’m very enthusiastic for them to stop worrying about issues such as blood pressure and cholesterol." (GP 53).
Prognosis and life expectancy	“And at the age of 86, you know, you wonder, if you do treat these, is it really going to make any difference? You know, people are, are maybe going to live until they’re, they’re 90 or something like that. So over the next four years, will it make any difference? And, you know, realistically, probably it won’t make much difference." (GP49) “I think we do need to manage their risk factors. It doesn’t matter if they’re old or not (…) people are living longer and they are very healthy” (GP50)
Balancing benefits and harms	"The main challenge is to, are you doing more harm or good by treating them. Are you going to cause them more issues by treating them, for example are you going to drop their blood pressure and they fall over and break their hip and they die in 6 months. Or are you better off leaving them alone and keeping your fingers crossed that they don't have a heart attack in 12 months, 2 years. It's very difficult." (GP51) “You can’t do much about their age now can you and, again you run the risk of overtreating them. And perhaps one of these things is whatever you do don’t do any harm (…) if you’ve got the healthy elderly who have maybe minor risk factors (…) rather than giving them drugs I think you’ve probably got to think about not harming them.” (GP23)
**Treatment complexity and optimising care**	
Perceived modifiability of CVD risk in older people	“People who have reached that other side of 75 you know they've got genes for longevity. I have got to assume this person will reach 95 and they're 75." (GP12) “I might go a little bit… strongly on the risk factors because they haven’t got much time to… on their hands to improve their cardiovascular risk factors. I might start them on medication sooner rather than later.” (GP39)
Less stringent treatment thresholds and targets	"I think you have to loosen up the targets quite frankly because I can get them to target but that then becomes a compliance thing because they just hate, they just, they just feel miserable" (GP51)
Polypharmacy	"[In] older people we have to be very, very careful of the dosage. It all depends upon their clinical state of health then. And we have to be very, very careful of the interaction of the medication and their cognition and understanding of the medication intake." (GP15)
Deprescribing	"I do a lot of work in palliative care. And I also have a lot of patients in retirement village and nursing homes and so on. And I encourage them, all those people, to stop the medications that are associated with prolonging life, such as statins and aspirin and, and so on. I think if people have got cognitive impairment and therefore have to be in an aged care facility, we shouldn’t be trying to avoid heart attacks. " (GP53) “I’m keen to try and reduce medication where I can if possible and would look at ways of trying to streamline it… minimise side effects of the different medications they may be on” (GP20).

*See S1 Table for evidence related to GPs’ decision making approach

### Applicability of primary CVD prevention guidelines for older people

Contradictory stances emerged regarding how GPs viewed the applicability of primary CVD prevention guidelines. Some GPs seemed to assume that guidelines are applicable to patients of all ages and because the use of absolute CVD risk (AR) is recommended in the Australian guidelines, and is available as a tool in practice software, presume it must have been validated for all ages. Other GPs were aware of limitations with the guidelines, for example, that Framingham based AR models cut off at age 74 and may therefore not be valid for patients aged 75+. Some GPs mentioned recent studies and/or guideline recommendations that specify adjusted treatment targets for older people, and other GPs stated that they were unsure about guideline recommendations for this population.

This variation was also reflected in management approaches. Several GPs described their approach to CVD risk management as largely the same in older versus younger patients, driven by the perceived high importance of reducing CVD risk (factors). Other GPs mentioned that their approach is generally *“less intense”* (GP55) in older people and/or described factors that they take into account in their decision making, in particular frailty, quality of life, balancing benefits and harms, multimorbidity, and prognosis or life expectancy. We will discuss each of these factors in more detail below.

### The older patient context

#### Fit versus frail older adults

Level of frailty played a more important role than age in determining how GPs manage CVD risk. GPs tended to take a similar approach to CVD risk management in patients regardless of age if the patient is fit and healthy. Frail patients, including patients in nursing homes, were less aggressively managed generally. Several GPs commented that CVD risk is less modifiable in frailer patients, due to inability to exercise or unfeasible treatment targets (see ‘Less stringent treatment thresholds and targets’ below). Although according to some, lifestyle change is still possible. Some GPs mentioned that it is important not to misclassify patients as frail or fit, as it cannot be presumed that these states are stable over time.

#### Quality of life

Several GPs expressed that their main goal of care is to improve/maintain older patients’ quality of life, mobility, independence and activities of daily living, and according to that perspective, managing CVD risk is an added bonus for some but a necessity for others. Some, but not all, GPs therefore expressed a preference for “prescribing” lifestyle change, in particular exercise, over medication as it has broader benefits for general quality of life including improved mobility, cognitive function, and falls risk management.

Similarly, patients may be encouraged to take medication if it is felt that medication may improve their quality of life (e.g. a CVD preventive medication might be more likely to be prescribed if it also helped alleviate a patient’s shortness of breath).

#### Multimorbidity

Several GPs commented that they are less concerned about high blood pressure and cholesterol in older patients with multimorbidity and they may decide not to manage CVD risk in these patients, as lifestyle change and medication may reduce already poor quality of life (e.g. due to polypharmacy and increased risk of drug related problems, or stopping enjoyable habits for limited health benefit).

#### Prognosis and life expectancy

Contrasting ideas about life expectancy can justify both decisions to manage and to not manage CVD risk. According to some GPs, as life expectancy is difficult to predict for an individual older patient, there is still scope for preventive management to make a difference. Older patients’ shorter life expectancy prompted some GPs to prescribe medication to try and achieve an effect as soon as possible.

Several GPs questioned the usefulness of preventive medication (in particular statins) for older patients if they are unlikely to live long enough to benefit from it. Estimated life expectancy was also weighed against the patient’s risk level with patients perceived to be at higher risk (e.g. very high blood pressure or high AR) more likely to be treated regardless of their prognosis. Some took CVD risk management as an opportunity to talk about end of life planning and advanced care plans, although others commented that their patients are not inclined to discuss these issues with their GP.

#### Balancing benefits and harms

GPs talked about the challenge of finding the appropriate balance between risk reduction, reduced quality of life (particularly due to side effects), and the burden of taking medication in the context of limited life expectancy. The point was raised by some GPs that certain patients might prefer a shorter life and a faster death due to a heart attack over living longer and dying slowly in a nursing home setting. At the same time, GPs mentioned the need to prevent stroke and especially stroke related disability in older people, resulting in a dilemma around the benefits and harms of preventive medication.

Falls risk was a commonly mentioned side effect that GPs want to avoid which influenced the decision to prescribe anti-hypertensive medication, and subsequent treatment dosage. Other side effects of medication mentioned included body pain with statins, and liver dysfunction.

### Treatment complexity and optimising care

#### Perceived modifiability of CVD risk in older people

Age was mentioned as one of the main CVD risk factors, thereby generally reducing the modifiability of CVD risk in older people: *"because as I said age itself is… harder to control"* (GP39). Often, a distinction was made between the ‘young-old’ (aged 75–85 years) and the ‘old-old’ (85 years plus) and several GPs commented that they would not start preventive treatment in the latter group.

At the same time, risk modifiability was considered to be influenced by patient context (e.g. general health, multimorbidity–see ‘The older patient context’ above) and patient motivation rather than age. The concept of some older adults being “genetically young” and showing a “degree of survivorship” was mentioned. This could either motivate GPs to actively manage risk or result in the perception that if CVD isn’t established at age 75, active risk management is not required.

Many GPs thought that risk was less modifiable with lifestyle change than medication.

However several also spoke of non-CVD risk related benefits to lifestyle change, and patients reducing their CVD risk through lifestyle changes even at an older age. Perceived modifiability also depended on older patients’ motivation to take medications or make lifestyle changes. Many GPs talked about older patients being change averse, specifically in regards to smoking cessation, diet, and exercise.

#### Less stringent treatment thresholds and targets

Although some mentioned using the same approach in older versus younger patients, many GPs commented that treatment targets and prescribing thresholds are not feasible for older people and need to be applied more flexibly due to increased risk of side effects, frequent polypharmacy, and the complexities of multimorbidity in this group. This flexibility was mentioned generally and specifically in regards to cholesterol, blood pressure, and diabetes control.

#### Polypharmacy

GPs mentioned age related differences in how the body responds to medication, and the increased risk of interactions between medication and various factors including other medication, cognition, and alcohol. One GP expressed a hesitation to introduce new medications, as it can be difficult to distinguish side effects of medication from multimorbidity or other health issues. Some GPs raised concerns about overtreating older patients who are at high risk due to their age but have otherwise minor risk factors.

#### Deprescribing

Some GPs also mentioned a desire to minimise medication burden by stopping, or not initiating medication, especially in the very elderly or those in a nursing home, or if the patient is already taking multiple medicines. Not starting, or stopping, was more often mentioned for cholesterol than blood pressure lowering medication. GPs were more likely to recommend stopping medication if they thought it would improve quality of life. Some GPs mentioned using absolute risk (e.g. showing a patient that high risk is mainly caused by older age) as an argument for avoiding unnecessary CVD medication and reducing polypharmacy.

#### Shared decision making issues

Patient preferences were mentioned often, in relation to decision making involvement and making a trade-off between potential benefits and harms. Some GPs perceived a preference for paternalism and the GP making the decision in some of their older patients. On the other hand, some GPs commented that patient preferences for involvement are personality not age-based. Personal, cultural, and family attitudes towards longevity and death were also raised as important factors when making decisions about preventive medication. Several GPs talked about the challenge of keeping patients on potentially unnecessary medication at the urging of family members or because it has been prescribed by specialists who are perceived to be more strict in their CVD risk management approach.

Although most GPs adapted their care for older people to some extent, some GPs seemed to be particularly good at tailoring to the older patient context. These GPs were more aware of the evidence around CVD risk assessment and management in older adults, were concerned about polypharmacy and balancing the harms and benefits of taking preventive medications, expressed the opinion that lifestyle change is achievable for older adults, tailored their lifestyle advice according to patients’ capabilities and preferences, and were keen to engage older adults and carers in decision making regarding interventions. However, it is important to note that often these GPs did not show all of these characteristics and, almost without exception, GPs expressed uncertainty around (some aspects of) CVD risk management in older people. Table 3 provides a summary of the shared decision making issues reported by GPs, with illustrative quotes from the data.

**Table 3:** Shared decision making issues related to GPs’ decision making about primary prevention of cardiovascular disease in older adults.

Shared decision making issues	Illustrative quote
Personality	"My elderlies really like being involved in decision making. They like being educated as to why I am suggesting something. I have one or two elderlies who are of the old school just want to do what the Doctor says but most of mine actually want to understand what's going on and want to be kept up to date. And a few of mine actually Google." (GP27)
Cultural values	"They have a different cultural attitude towards longevity and that is you squeeze every minute out of life. And you have to respect that. And, so there’s no question that you’d continue with Lipitor and aspirin, even in people with dementia" (GP53)
Acceptance of death	"I have some patients if they, that are (…) in that over 75 age bracket that say look I’ve got to die of something, stop trying to keep me alive, stop giving me all these medications." (GP42)
Conflict with other health professionals	"Older patients that you've known for 20 plus years tend to want you to be paternalistic and tend to trust you….they’re telling you they don't want to take it but and the cardiologist is saying they must take it and then they want you to (…) give them permission to not take it (…). And that's a very hard path to tread." (GP12)
Conflict with family	“It is interesting, however, the number of relatives that object to mum with metastatic breast cancer being, having her Lipitor stopped.” (GP53)
Experience of harms	"I do listen to patient priorities and preferences. If the cholesterol medication makes them feel miserable and icky (…) then it's really not worth them going through that in terms of quality of life for a few percentage points on a risk tool." (GP42)

## Discussion

This study identified factors that are likely to contribute to variation in how GPs manage CVD risk in older people (defined as ≥75 years). This includes: differences in GP knowledge of available evidence for CVD prevention, applicability of the guidelines for older people, attitudes towards ageing, awareness of older patient context, and perceived importance of CVD prevention and risk reduction in the older population. Some GPs suggested treating older patients the same as younger patients, assuming the guidelines apply equally to patients of all ages; other GPs considered older people’s management required careful adjustment according to their specific context and circumstances. These latter GPs often mentioned the importance of reducing the burden of treatment and prioritising care (often with prevention perceived as less important than symptom management and improving/maintaining quality of life). It is not surprising that there is substantial variation in GP practice. Two recent reviews independently showed that clinical practice guidelines are highly disease specific and tend to ignore patient context [28], often recommending the initiation of preventive medication therapy without consideration of multimorbidity, advanced illness, or limited life expectancy [25].

When discussing CVD risk management approaches, GPs often distinguished between fit versus frail older patients. Many GPs treated healthy and fit older people the same as younger patients but were more cautious in their approach with frail older people and would consider not starting, or stopping, preventive medication in this group. GPs appeared to differ in their definitions of frailty although many referred to the following factors: multimorbidity, taking multiple medicines, dependence/living in nursing home, life limiting illness (for example cancer), and dementia [38]. Fit older patients were often defined as the ‘genetically young,’ on few medications, independent, and with an active lifestyle. Current clinical evidence seems to concur with this approach; increasingly studies show that although treatment with statins and/or hypertension medication might be beneficial in older patients who are healthy and functionally independent, there is uncertainty about the benefits and increased risk of harm in frail older patients (including those with multimorbidity and/or a limited life expectancy) [2, 39, 40].

The potential benefits and harms of preventive treatment for older people at high risk of a first primary CVD event also apply to those with prior history of CVD; and the probability of either of these classifications increases with age (along with prevalence of undiagnosed ‘silent’ events [41] which carry a similar prognosis to clinical events–these are especially likely where there is a history of clinical CVD or a person is at high risk of a first primary event [42]). A qualitative focus group study of secondary CVD prevention in older people found similar dilemmas and uncertainties about the potential benefits and harms of secondary cardiovascular prevention, with management often based on frailty rather than chronological age [43].

When discussing CVD risk management in older people, GPs almost exclusively talked about patients at either end of the spectrum (fit/robust versus frail), whereas in practice they will see many people who are likely to fit somewhere in the middle. A systematic review of 21 studies in 61,500 older people in the community estimated the prevalence rate of frailty as 9·9% (95% CI 9·6–10·2), and increasing with age (up to 26% in those 85 years or older) [44]. Similarly, more than 60% of people 65 and over have two or more comorbidities, and the number of comorbidities increases rapidly with age [45]. This may indirectly imply uncertainty about the best course of action for the group of patients who could not easily be identified as fit or frail. Dalton et al. [9] reported that clinicians generally find it straightforward to provide recommendations about colorectal cancer screening for older patients who are in good health (screening recommendation) or poor health (recommendation not to screen) but were uncertain about their recommendations for patients with moderate morbidities. Clinicians considered it more challenging to estimate life expectancy and weigh the potential benefits and downsides of screening for this group. This suggests that the issues are relevant to the management of older patients more broadly.

Our results suggest that some GPs make a subjective assessment of frailty on their older patients and this in turn influences the approach they take on primary CVD prevention. More formal measures of frailty [46, 47] have been shown to be predictive of mortality, but it remains to be seen whether more formal objective assessments of frailty could be used to guide decisions about primary CVD prevention.

Due to the close balance between benefits and harms, shared decision making has been promoted in primary CVD prevention for older people [25, 40]. However, in line with previous work [48], our interviews suggest that few GPs use a shared decision making approach and many refer to challenges associated with older patient involvement, including: third party involvement, a perception that older patients prefer a paternalistic relationship with their GP, and cognitive impairment. This is somewhat in contrast with a focus group study on secondary CVD prevention in which the majority of GPs mentioned using shared decision making as a way to deal with uncertainties around the management of older people [43]. The majority of the GPs in this study were highly skilled GPs (e.g. GPs with an interest in geriatrics and academic GPs), which may explain part of this difference.

The strengths of this study include a heterogeneous sample with a wide range of characteristics. Since GPs with strong opinions may have been more likely to agree to participate, some selection bias is possible. However, diverse opinions and approaches were reported, and on recruitment participating GPs were not aware that the interviews focused on CVD risk management in older patients specifically, suggesting that strong selection bias is unlikely. The sampling strategy aimed to maximise the range of experiences and views rather than be representative of the general GP population. Future research could investigate differences between GP and patient perceptions by comparing GP-patient dyads.

## Conclusions

Depending on their perceptions of ageing and CVD prevention, and their knowledge of available evidence, GPs vary in how they make decisions about primary CVD prevention in older people. More support for SDM with older patients in GP practice (e.g. training, decision aids) is needed. In addition, guidelines should adopt an approach that is more suited to the increasing population of older people with multimorbidity. These guidelines should provide guidance on the challenges faced by GPs, including limited available evidence, and balancing the harms and benefits of CVD prevention in older people of varying life expectancy and levels of frailty.

## Supporting Information

S1 Text Cardiovascular Disease risk assessment interview study. Semi-structured interview schedule.
(DOC)

S1 Table Evidence relating to GPs’ decision making approach about primary prevention of cardiovascular disease in older adults. Evidence relating to GPs’ decision making approach about primary prevention of cardiovascular disease in older adults as described by Table 2 (main text).
(DOCX)
