# Supplementary material for: General Practitioners’ Decision Making about Primary Prevention of Cardiovascular Disease in Older Adults: A Qualitative Study
Source: PLoS One. 2017 Jan 13;12(1):e0170228. doi: 10.1371/journal.pone.0170228 (PMC5234831; doi:10.1371/journal.pone.0170228)
Supplement: S1 Text — Semi-structured interview schedule. (DOC) [file pone.0170228.s001.doc]

**General practitioners' decision making about primary prevention of cardiovascular disease in older adults: A qualitative study – S1 Text**

**Cardiovascular Disease risk assessment interview study**

**CLINICIAN INTERVIEWS (75+)**

**___________________________________________________________________________**

**PART 1: Questions about Risk Assessment in the general population**

1. When you assess cardiovascular risk for a patient, how do you do it? (prompt: what do you do at each step in this procedure?)
2. Do you use absolute risk scores or not? (if not mentioned)
   1. Why do/don’t you like to use this approach? Please explain your concerns about the absolute risk approach (if AR not used)
   2. What do you prefer to use instead? (if AR not used)

**PART 2: Questions about CVD Risk Assessment among patients 75+**

1. Among an older patient population (i.e. 75 years or older) how do you decide whether to assess a patient’s cardiovascular risk?
   1. How is this different from younger patients? (prompt if approach not different –to replace b) & c): How do you handle patient factors that are more common with increased age such as competing health problems or shorter life expectancy?)
   2. Which factors do you take into account in your decisions about whether or not to assess cardiovascular risk among older patients?
   3. Are there any other ‘patient cues’ that you use to decide whether to assess cardiovascular risk in an older patient? (prompt: how much do you consider non-clinical patient characteristics? For example co-morbidities, life expectancy, functional status, QoL, patient priorities/preferences etc).
2. When you assess cardiovascular risk among older patients, how do you do it?
   1. What are the differences compared to how you assess cardiovascular risk in the general population?
3. If AR is used: What are your thoughts about *absolute* risk assessment for patients over 75 years compared to those under 75 years? (if not mentioned)
   1. Please explain your ideas/concerns about the absolute risk approach (if AR not used) among older patients (prompt: can you give me an example?)
   2. If not mentioned: Are there any restrictions that you are aware of for using an absolute risk approach amongst patients who are 75 years or older? (prompt: how do you deal with this restriction?).
4. If AR is not used: What are your thoughts about cardiovascular disease risk assessment for patients over 75 years compared to those under 75 years? (if not mentioned)
   1. Please explain your ideas/concerns about CVD risk assessment among older patients (prompt: can you give me an example?)

**PART 3: Questions about Risk Management among patients 75+**

7. After assessing risk, the next consideration is how to manage it. Can you tell me about your approach to *managing* cardiovascular risk for older patients?

1. What are the differences compared to how you manage/treat cardiovascular risk in the general population? (prompt if approach not different –to replace b) & c): how do you handle patient factors that are more common with increased age such as competing health problems or shorter life expectancy?)
2. Which factors do you take into account when deciding how to manage/treat cardiovascular risk among older patients?
3. Are there any other ‘patient cues’ that you use to decide if and how you are going to manage/treat risk factors in an older patient? (prompt: how much do you consider patient characteristics that are less related to CVD? For example co-morbidities, life expectancy, functional status, QoL, patient priorities/preferences etc).
4. Does your approach to prescribing medication for CVD risk vary in older versus younger patients? If yes how? (if not mentioned)
5. How do you advise about lifestyle changes in older versus younger patients (if not mentioned)?

8. How modifiable do you feel cardiovascular risk is in older patients compared to younger patients (if not mentioned)?

1. Does this differ for lifestyle changes versus drug treatment?

9. In your experience, is there any difference in patients’ attitudes towards taking drugs to reduce their CVD risk in older versus younger patients (if not mentioned)?

1. What about age differences in treatment adherence?

**PART 5: Questions about guideline recommendations for patients 75+**

10. How do you think current guidelines for cardiovascular risk relate to adults who are 75 years or older? (Prompt: are you aware of any specific CVD guidelines or guideline recommendations for patients who are 75 years or older?)

1. How could this be improved?

*Note: when the new NVDPA guidelines are released add a question about the recommendations for patients 5+. E.g. “For those GPs who use AR: The new NVDPA absolute cardiovascular disease risk guidelines recommend …..”*

11. How do you find involving older patients in the decision making process about cardiovascular risk assessment and management?

**PART 6: scenarios**

***Notes for interviewer:***

- *If GPs can’t use the information in the format provided, ask about these scenarios in a more general way – e.g. how do you manage patients if they have one elevated risk factor, such as blood pressure, but their overall risk isn’t high enough to start treatment?*
- *Specify AR if they say they would calculate the risk*

12. Please describe how you would manage the following cases:

1. Raised absolute risk but no elevated risk factors: *Male patient, aged 86 years old, no history of diabetes, no family history of CVD, smoker, Systolic BP 129, total cholesterol 5.2, HDL cholesterol 1.5, cholesterol ratio TC/HDL 3.6. (note: absolute risk 20% for 74 year old)*
2. Raised absolute risk and medium elevat*ed risk factors (below threshold): Male patient, aged 86 years old, no history of diabetes, no family history of CVD, non-smoker, Systolic BP 145, total cholesterol 6.0, HDL cholesterol 1.0, cholesterol ratio TC/HDL 6.0 (note: absolute risk 22% for 74 year old)*

13. What are the main challenges in cases such as these? (prompt: where the overall risk is raised but individual risk factors are low to moderate)

1. (If time): Would you change your management of the cases we just discussed if the patients were 96 years old instead of 86? And would there be any differences if they were 76 years old?
2. Do you have any concerns or ideas about assessing and managing cardiovascular risk among older patients that you haven’t mentioned before?
