# Supplementary material for: General Practitioners’ Decision Making about Primary Prevention of Cardiovascular Disease in Older Adults: A Qualitative Study
Source: PLoS One. 2017 Jan 13;12(1):e0170228. doi: 10.1371/journal.pone.0170228 (PMC5234831; doi:10.1371/journal.pone.0170228)
Supplement: S1 Table — Evidence relating to GPs’ decision making approach about primary prevention of cardiovascular disease in older adults as described by Table 2 (main text). (DOCX) [file pone.0170228.s002.docx]

**General practitioners' decision making about primary prevention of cardiovascular disease in older adults: A qualitative study – S1 Table**

S1 Table. Evidence relating to GPs’ decision making approach about primary prevention of cardiovascular disease in older adults as described in Table 2 (main text).

| **Approach** | **Related evidence** |
| --- | --- |
| **Applicability of primary CVD prevention guidelines for older people** | |
| Same guidelines for all ages | Modelling work suggests minimal difference in potential life years gained with treatment for younger vs older people who are at the same 5-year CVD risk [1]. |
| No valid guidelines for older adults | Data to derive FHS risk and QRISK models based on individuals <75 years [2-4], data to derive QRISK lifetime model included individuals up to 84 years [5]. Guidelines exist for use of both statins [6] and blood pressure lowering treatment [7] in older people. |
| Different guidelines for older adults | Major U.S. guidelines have relaxed systolic BP target for treatment to 150 mmHg [8], however there is substantial disagreement on whether there is an evidence base for this [8]. |
| **The older patient context** | |
| Fit versus frail older adults | Randomised controlled trial evidence in the elderly supports treatment to lower blood pressure and cholesterol in healthy older people. There is limited evidence on the effects of these treatments in frail older people, but analysis of one trial suggests that treating hypertension is still beneficial for frail older patients [9]. Some guidelines specifically recommend different management of cardiovascular disease prevention for ‘fit’ and ‘frail’ older patients [6, 10, 11]. Additionally, the instability feature of frailty may make it difficult to identify which patients are actually frail [12]. |
| Quality of Life (QoL) as treatment goal | In general, trial data in elderly people has found that pharmacologic treatment to lower blood pressure had no negative impact on QoL, or produced some improvement in QoL [13]. |
| Multimorbidity | Most clinical practice guidelines (CPGs) do not discuss their recommendations in terms of elderly patients with multiple comorbidities. If the applicable CPGs are followed for all comorbidities, treatment may cause a considerable burden to patients and caregivers, as well as other adverse effects [14]. |
| Prognosis and life expectancy | Trial data in older aged people demonstrates statistically significant reduction of cardiovascular disease after 3.2 years statin treatment to lower cholesterol [15] and after 2 years with treatment to lower blood pressure [16]. Modelling work suggests minimal difference in potential life years gained with cardiovascular preventative treatment for younger vs older people [1]. |
| Balancing benefits and harms | The evidence is far more limited in older patients, making the benefits and harms of intervention difficult to estimate. The GRADE approach to evidence-based medicine and shared decision making suggests that considering patient values is particularly important when benefits do not clearly outweigh harms [17]. |
| **Treatment complexity and optimising care** | |
| Perceived modifiability of CVD risk in older people | For Australians aged 65 years, expected further life expectancy is 19 years in men and 22 years in women. For Australians aged 85 years, expected further life expectancy is 5.9 years in men and 7.1 years in women [6]. |
| Less stringent treatment thresholds and targets | The reviews of trials of more versus less intensive blood pressure reduction also do not provide a clear picture. Wald and Law [18] found that combinations were more effective in lowering blood pressure, but the interpretation of impact on clinical events is confounded by drug class combinations. Susantitaphong's 2013 analysis for patients with CKD found small and non-significant effects favouring combinations, but with higher adverse effect rates. |
| Polypharmacy | The use of more drugs than are clinically indicated is common among frail older adults [7]. Polypharmacy is associated with increased risk of adverse events. |
| Deprescribing | There is limited direct evidence from clinical trials to be able to answer the question about whether deprescribing cardiovascular drugs is beneficial or harmful in older people. A recent clinical trial found that stopping statin was safer and may be associated with improved quality of life in people with advanced life limiting illness. |

**References**

1. Liew SM, Jackson R, Mant D, Glasziou P. Should identical CVD risks in young and old patients be managed identically? Results from two models. BMJ Open. 2012;2(2): e000728.

2. D’Agostino RB, Vasan RS, Pencina MJ, Wolf PA, Cobain M, Massaro JM, et al. General cardiovascular risk profile for use in primary care The Framingham Heart Study. Circulation. 2008;117: 743-753.

3. Pencina MJ, D'Agostino RB, Sr., Larson MG, Massaro JM, Vasan RS. Predicting the 30-year risk of cardiovascular disease: the Framingham Heart Study. Circulation. 2009;119(24): 3078-3084.

4. Hippisley-Cox J, Coupland C, Vinogradova Y, Robson J, Minhas R, Sheikh A, et al. Predicting cardiovascular risk in England and Wales: prospective derivation and validation of QRISK2. BMJ. 2008;336(7659): 1475-1482.

5. Hippisley-Cox J, Coupland C, Robson J, Brindle P. Derivation, validation, and evaluation of a new QRISK model to estimate lifetime risk of cardiovascular disease: cohort study using QResearch database. BMJ. 2010;341: c6624.

6. Hamilton-Craig I, Colquhoun D, Kostner K, Woodhouse S, d’Emden M. Lipid-modifying therapy in the elderly. Vasc Health Risk Manag. 2015;11: 251-263.

7. Benetos A, Rossignol P, Cherubini A, Joly L, Grodzicki T, Rajkumar C, et al. Polypharmacy in the aging patient: management of hypertension in octogenarians. JAMA. 2015;314(2): 170-180.

8. James PA, Oparil S, Carter BL, Cushman WC, Dennison-Himmelfarb C, Handler J, et al. 2014 evidence-based guideline for the management of high blood pressure in adults: report from the panel members appointed to the Eighth Joint National Committee (JNC 8). JAMA. 2014;311(5): 507-520.

9. Warwick J, Falaschetti E, Rockwood K, Mitnitski A, Thijs L, Beckett N, et al. No evidence that frailty modifies the positive impact of antihypertensive treatment in very elderly people: an investigation of the impact of frailty upon treatment effect in the HYpertension in the Very Elderly Trial (HYVET) study, a double-blind, placebo-controlled study of antihypertensives in people with hypertension aged 80 and over. BMC Med. 2015;13: 78.

10. Mancia G, Fagard R, Narkiewicz K, Redon J, Zanchetti A, Bohm M, et al. 2013 ESH/ESC guidelines for the management of arterial hypertension: the Task Force for the Management of Arterial Hypertension of the European Society of Hypertension (ESH) and of the European Society of Cardiology (ESC). Eur Heart J. 2013;34(28): 2159-2219.

11. Aronow WS, Fleg JL, Pepine CJ, Artinian NT, Bakris G, Brown AS, et al. ACCF/AHA 2011 expert consensus document on hypertension in the elderly: a report of the American College of Cardiology Foundation Task Force on Clinical Expert Consensus Documents developed in collaboration with the American Academy of Neurology, American Geriatrics Society, American Society for Preventive Cardiology, American Society of Hypertension, American Society of Nephrology, Association of Black Cardiologists, and European Society of Hypertension. J Am Soc Hypertens. 2011;5(4): 259-352.

12. Clegg A, Young J, Iliffe S, Rikkert MO, Rockwood K. Frailty in elderly people. Lancet. 2013;381(9868): 752-762.

13. Soni RK, Porter AC, Lash JP, Unruh ML. Health-related quality of life in hypertension, chronic kidney disease, and coexistent chronic health conditions. Adv Chronic Kidney Dis. 2010;17(4): e17-26.

14. Boyd CM, Darer J, Boult C, Fried LP, Boult L, Wu AW. Clinical practice guidelines and quality of care for older patients with multiple comorbid diseases: implications for pay for performance. JAMA. 2005;294(6): 716-724.

15. Shepherd J, Blauw GJ, Murphy MB, Bollen ELEM, Buckley BM, Cobbe SM, et al. Pravastatin in elderly individuals at risk of vascular disease (PROSPER): a randomised controlled trial. Lancet. 2002;360(9346): 1623-1630.

16. Beckett NS, Peters R, Fletcher AE, Staessen JA, Liu L, Dumitrascu D, et al. Treatment of hypertension in patients 80 years of age or older. NEJM. 2008;358(18): 1887-1898.

17. Guyatt GH, Oxman AD, Santesso N, Helfand M, Vist G, Kunz R, et al. GRADE guidelines: 12. Preparing summary of findings tables-binary outcomes. J Clin Epidemiol. 2013;66(2): 158-172.

18. Wald DS, Law M, Morris JK, Bestwick JP, Wald NJ. Combination therapy versus monotherapy in reducing blood pressure: meta-analysis on 11,000 participants from 42 trials. Am J Med. 2009;122(3): 290-300.

19. Susantitaphong P, Sewaralthahab K, Balk EM, Eiam-ong S, Madias NE, Jaber BL. Efficacy and safety of combined vs. single renin-angiotensin-aldosterone system blockade in chronic kidney disease: a meta-analysis. Am J Hypertens. 2013;26(3): 424-441.
